# Thirty-Two Complete Genome Assemblies of Nine *Yersinia* Species, Including *Y. pestis*, *Y. pseudotuberculosis*, and *Y. enterocolitica*

**DOI:** 10.1128/genomeA.00148-15

**Published:** 2015-04-30

**Authors:** Shannon L. Johnson, Hajnalka E. Daligault, Karen W. Davenport, James Jaissle, Kenneth G. Frey, Jason T. Ladner, Stacey M. Broomall, Kimberly A. Bishop-Lilly, David C. Bruce, Susan R. Coyne, Henry S. Gibbons, Chien-Chi Lo, A. Christine Munk, C. Nicole Rosenzweig, Galina I. Koroleva, Gustavo F. Palacios, Cassie L. Redden, Yan Xu, Timothy D. Minogue, Patrick S. Chain

**Affiliations:** a Los Alamos National Laboratory (LANL), Los Alamos, New Mexico, USA; b United States Army Medical Research Institute of Infectious Diseases (USAMRIID) Diagnostic Systems Division (DSD), Fort Detrick, Maryland, USA; c Naval Medical Research Center (NMRC)-Frederick, Fort Detrick, Maryland, USA; d Henry M. Jackson Foundation, Bethesda, Maryland, USA; e Center for Genome Sciences (CGS), United States Army Medical Research Institute of Infectious Diseases (USAMRIID), Fort Detrick, Maryland, USA; f United States Army Edgewood Chemical Biological Center (ECBC), Aberdeen Proving Ground, Aberdeen, Maryland, USA

## Abstract

The genus *Yersinia* includes three human pathogens, of which *Yersinia pestis* is responsible for >2,000 illnesses each year. To aid in the development of detection assays and aid further phylogenetic elucidation, we sequenced and assembled the complete genomes of 32 strains (across 9 *Yersinia* species).

## GENOME ANNOUNCEMENT

The genus *Yersinia* contains 11 species, with three human pathogens, *Y. pestis*, *Y. pseudotuberculosis*, and *Y. enterocolitica*. Of these, *Y. pestis* is the most virulent, causing >2,000 global cases of plague annually, along with three global pandemics (1, 2). *Y. pestis* is a category A pathogen and potential biowarfare agent (3, 4), while *Y. pseudotuberculosis* and *Y. enterocolitica* cause food-borne self-limiting enteric diseases with low mortality rates (5). Recently, the list of strains for consideration in diagnostic assay development was released by the Association of Analytical Communities (AOAC) International, including strains that should be recognized (inclusivity) and ignored (exclusivity) by the assays (6). Here, we present the completed genome assemblies for 32 (see Table 1) of the 33 listed *Yersinia* strains (YPNN7 *Y. pseudotuberculosis* IB was not included due to technical issues).

**TABLE 1:** List of strains included in the data set, their accession numbers, and plasmids

Strain name	AOAC	Panel^a^	Accession no.	Size (Mb)	No. of indicated plasmid			
					pPCP	pCD/pYV	pMT	Other
*Y. aldovae*								
670-83	YPNN17	E	CP009781	4.47				
*Y. enterocolitica*								
2516-87	YPNN13	E	CP009837, CP009838	4.60		1		
8081	YPNN12	E	CP009845, CP009846	4.68		1		
WA	YPNN11	E	CP009366, CP009367	4.61		1		
*Y. frederiksenii*								
Y225^b^	YPNN15	E	CP009363, CP009364	4.55				1
*Y. intermedia*								
Y228	YPNN16	E	CP009801	4.85				
*Y. kristensenii*								
Y231	YPNN14	E	CP009997	4.49				
*Y. pestis*								
A1122	YP12	I	CP009839–CP009841	4.67	1		1	
Angola	YP7	I	CP009934– CP009937	4.67	1	1	1	
Antiqua	YP3	I	CP009903– CP009906	4.88	1	1	1	
CO92 pgm-	YP1	I	CP009971– CP009973	4.72		1	1	
Dodson	YP15	I	CP009842– CP009844	4.77		1	1	
El Dorado	YP16	I	CP009782– CP009785	4.83	1	1	1	
Harbin35	YP9	I	CP009701– CP009704	4.70	1	1	1	
Java9^c^	YP11	I	CP009992– CP009996	4.82	1	1		2
KIM5	YP2	I	CP009833– CP009836	4.78	1	1	1	
Nairobi	YP8	I	CP010293, CP010294	4.47	1			
Nicholisk 41	YP13	I	CP009988– CP009991	4.70	1	1	1	
PBM19	YP10	I	CP009489– CP009492	4.86	1	1	1	
Pestoides B	YP4	I	CP010020– CP010023	4.79	1	1	1	
Pestoides F	YP5	I	CP009713– CP009715	4.72		1	1	
Pestoides G	YP6	I	CP010246– CP010248	4.73		1	1	
Shasta	YP14	I	CP009721– CP009724	4.83	1	1	1	
*Y. pseudotuberculosis*								
1	YPNN10	E	CP009786	4.72				
EP2/+	YPNN8	E	CP009758, CP009759	4.77		1		
IP32953	YPNN4	E	CP009710– CP009712	4.83		1		1
MD67	YPNN9	E	CP009757	4.72				
Pa3606	YPNN6	E	CP010067– CP010069	4.83		1		1
PB1/+	YPNN3	E	CP009779, CP009780	4.76		1		
YPIII	YPNN5	E	CP009792	4.68				
*Y. rohdei*								
ATCC 43380	YPNN2	E	CP009787	4.37				
*Y. ruckeri*								
YRB	YPNN1	E	CP009539	3.60				

a Refers to the AOAC listing (6) of either inclusivity (I) or exclusivity (E) strains.

b The plasmid in *Y. frederiksenii* is cryptic.

c The two plasmids listed as “other” for *Y. pestis* JAVA9 are pJARS35 and pJARS36.

Each genome was assembled using at least two data sets (specific data types and coverages are listed in the NCBI records), from Illumina (short- and/or long-insert paired data), Roche 454 (long-insert paired data), and/or PacBio long reads. The short- and long-insert paired data were assembled together in both Newbler and Velvet and computationally shredded into 1.5-kbp overlapping shreds. If the PacBio coverage was ≥100×, the data were assembled using the PacBio Hierarchical Genome Assembly Process (HGAP) (7). All data were additionally assembled in AllPaths (8). The consensus sequences from both HGAP and AllPaths were computationally shredded into 10-kbp overlapping pieces. All shreds were integrated using Phrap. Possible misassemblies were corrected and repeat regions verified using in-house scripts and manual editing in Consed (9–11). All genomes were assembled to finished-quality completion (12), and each assembly was annotated using an Ergatis-based (13) workflow, with minor manual curation.

The genome sizes averaged 4.68 ± 0.04 Mb (Table 1; the smallest is *Yersinia ruckeri* YRB, at 3.6 Mb, and the largest is *Y. pestis* Antiqua, at 4.9 Mb), with up to 4 plasmids (average, 1.6 ± 0.2). Each genome contains 3,161 to 4,419 coding sequences (average, 4,155 ± 39.9) and a G+C content of 47 to 48%. As many of the virulence genes are located on plasmids, it is interesting to note that of the 16 *Y. pestis* strains, only 9 had all three “traditional” plasmids (pYV/pCD1 [virulence/calcium dependence], pPCP [plasminogen activator], and pMT [murine toxin]), with one strain (*Y. pestis* Nairobi) containing the pPCP plasmid only.

### Nucleotide sequence accession numbers.

The GenBank accession numbers for all 32 genomes are listed in Table 1.
